# Supplementary material for: MetaRibo-Seq measures translation in microbiomes
Source: Nat Commun. 2020 Jun 29;11:3268. doi: 10.1038/s41467-020-17081-z (PMC7324362; doi:10.1038/s41467-020-17081-z)
Supplement: Supplementary file 10 — Supplementary Data 7 [file 41467_2020_17081_MOESM10_ESM.zip › File2/Confidence_High_Taxonomy/157075_out.krona.html]

Javascript must be enabled to view this page.

members
magnitude
magnitudeUnassigned
count
unassigned
taxon
rank

157075\_out

12

2
superkingdom
7

201174
phylum
1

class
1760
1

85011
order
1

1
family
2062

1883
genus
1


SRS063518\_contig\_number\_20727
species
164115
1

6
1239
phylum

class
186801
2

order
186802
2

1

SRS076929\_contig\_number\_23902
species
1898207

1
1950935

SRS042966\_contig\_number\_12097
species

4
1262999

SRS013638\_contig\_number\_15399SRS077392\_contig\_number\_114SRS143466\_contig\_number\_23148SRS971275\_contig\_number\_37232
species


SRS019068\_contig\_number\_contig-100\_11230.480839SRS024435\_contig\_number\_contig-100\_11625.178048SRS049995\_contig\_number\_21307SRS144135\_contig\_number\_contig-100\_13591.110980SRS144506\_contig\_number\_contig-100\_22650.208311
5
